# Use of public primary care facilities, economic development, and the health service transition

**DOI:** 10.7189/jogh.16.04142

**Published:** 2026-04-10

**Authors:** Krishna D Rao, Austin Schmidt, Yifeng Zhao

**Affiliations:** Department of International Health, Johns Hopkins University, Baltimore, USA

## Abstract

**Background:**

Many low- and middle-income countries (LMICs) have large networks of public primary care facilities (PCF) to provide affordable and quality health services close to communities. Public PCFs are expected to serve as the principal source of primary care. This study documents the extent to which public PCFs are used for illnesses treatable at the primary care level, and investigates the association between public PCF use, economic development, and UHC achievement.

**Methods:**

A cross-section of Demographic and Health Surveys conducted after 2014 in 46 LMICs were analysed. The sources of medical advice for children seeking care outside their home for the following illnesses were identified – acute respiratory infections (ARI), diarrhoea, and fever.

**Results:**

There is considerable between-country variation in utilisation of public PCFs; in most countries public PCFs received less than half the patients seeking medical advice for conditions treatable at the primary care level. Second, economic development is associated with a ‘health service transition’ characterised by two related trends – decline in the share of patients seeking medical advice at public providers overall and at public PCFs, and a proportionate increase in the share of patients seeking medical advice at private providers; use of public PCFs declined by around 24 percentage points between the average low-income and middle-income country. However, most of the between-country variation in public PCF use was due to factors other than income. Third, cross-country regression analysis indicated that public PCF use was not associated with UHC achievement because a similar range of services are offered by private providers. Public PCF use was associated with lower catastrophic health expenditures.

**Conclusions:**

The changes in care-seeking patterns and use of public PCFs brought about by economic development makes it critical to re-think primary health care service delivery models and financial protection mechanisms in transition countries.

Providing health services close to where people live has been a longstanding policy objective in many low- and middle-income countries (LMICs). To achieve this governments have built large networks of publicly financed, staffed, and resourced primary care facilities (public PCF) close to communities to deliver a range of public health, and personal preventive and curative health services. Public PCFs vary in size, staffing and services provided; they are typically ‘clinics’ comprised of a few rooms located in or near rural or urban communities. Their staffing depends on their type and level of public PCF, and usually comprise a combination of medical doctor, mid-level provider, nurse, pharmacist, laboratory technician, and affiliated community health workers. Governments spend a substantial part of their health budgets on PCFs; estimates indicate that on average between 33 to 36% (excluding governance and administration costs) of domestic government health expenditures is spent on primary health care [1].

Public PCFs are expected to serve as the main source of health services, *i.e.* personal preventive and curative health services of their catchment communities. However, this is not the case in many LMICs. Variable caseloads at health centres are reported in sub-Saharan countries - from 1.4 outpatient visits per day in Nigeria, 5.2 per day in Madagascar, 6 per day in Uganda, to 38 in Mozambique [2,3]. It is well known that in many LMICs private primary care providers are a significant source of curative care, though not for preventive services or institutional deliveries [4, 5]. A study of treatment seeking for children with fever/ARI in 12 low-income countries in Asia and sub-Saharan Africa, reported that in only two countries (Zambia and Senegal) at least half of the visits were to a public PCF; in Bangladesh it was around 5% [6]. A study from one state in India reported that only 4% of curative care visits were to the local public PCF, and even in villages where the public PCF was located, no more than 20% of outpatient visits were made to the public PCF, which declined with increasing distance [7].

The reasons for variable public PCF use are many. The Anderson behavioural model of health services use provides a useful lens to examine drivers of public PCF use. The model identifies contextual and individual predisposing, enabling, and need factors that facilitate or impede health service use. Studies on provider choice and public PCF bypassing report that use of public PCFs is influenced by predisposing (process and structural quality of care, availability of drugs, cultural beliefs, preference for private or higher-level providers), enabling (economic status, education, cost of health services, distance from health facility), and need (illness severity, having a chronic condition) factors [7–11]. Economic development and its accompanying transformations, such as, expansion of road networks, urban spread, and growth in the private health sector also influence care seeking patterns. These factors can affect demand for public PCF services and local health care markets. Grépin (2016) using data sets similar to this study notes that the transition from low-income to middle-income status is accompanied by increased private sector use, which then declines in upper-middle income countries presumably because of expansion of social safety nets and government investment in universal health coverage programmes [4]. However, the relationship between economic development and public PCF use may not be that straightforward – one study from India reported that increased road density was associated with greater institutional deliveries and ante-natal care; importantly, as road availability increased, patients switched from private providers to public PCFs and hospitals for institutional deliveries, and to public PCFs for ante-natal care [12].

This paper has three aims. First, to document the use of public PCFs in LMICs for curative services. Second, to examine how use of public PCFs varies with national income. Third, to examine the association between public PCF use, financial protection, and the achievement of universal health coverage (UHC). We examined PCF use from the perspective of curative primary care services to individuals. Specifically, we focussed on sources of medical advice for children seeking treatment for three tracer conditions – acute respiratory infections (ARI), diarrhoea, and fever. These tracer conditions were included because information on sources of medical advice for illness was only available for these conditions in the Demographic and Health Surveys, on which our analysis is based. As such, household preferences for providers treating their sick children are a good indicator of the household usual source of health care.

## METHODS

To achieve the study objectives, a cross-section of LMIC countries was compared on the outcomes of interest – namely, sources for medical advice for children will ARI, diarrhoea, or fever. The World Bank classification of countries by national income was used to identify LMIC countries eligible for inclusion in the study [13].

### Data sources

Data for this study was sourced from various Demographic and Health surveys (DHS) (Appendix 1 in the **Online Supplementary Document**). Demographic and Health surveys surveys are household survey where a probabilistic sample of households are selected that is representative of the national population. In the selected households the survey records if any children under five years of age had symptoms of diarrhoea, ARI, or fever in the two weeks prior to the survey and sought outpatient medical advice outside their home.

Because DHS surveys use standardised questionnaires and survey design, they allow for cross-country comparisons. DHS surveys have been conducted in 86 of the135 LMICs. From this universe we excluded 40 countries which did not have a DHS survey since 2014 or lacked data on the selected indicators. We selected 2014 as the cut off so that the surveys were relatively recent. All countries that had data on at least one of the childhood illness indicators (ARI, diarrhoea, fever) were included. The final sample included 45 countries which had data for ARI and diarrhoea, and 33 for fever (Figure S1 in the **Online Supplementary Document**). The survey years in the final sample ranged from 2014 to 2022. A total sample of 656 680 children were observed across 45 countries.

Information on selected indicators from the DHS surveys were abstracted using StatCompiler, an online tool provided by DHS to extract information on key indicators from DHS surveys [14]. To the best of our knowledge, no imputation is made for missing data by StatCompiler. All estimates are representative of the population; StatCompiler incorporates sampling weights to account for different selection probabilities of units. StatCompiler estimates were cross-checked with published DHS reports.

Information on per capita GDP and other country indicators was abstracted from the World Bank’s World Development Indicators database [15]. Information on primary school completion rates was sourced from the UNESCO Institute for Statistics [16]. To exclude the effects of COVID on income and health expenditure, countries with a DHS from 2020–2022 (n = 15) used 2019 income and expenditure data. Information on catastrophic health expenditures (CHE) and universal health coverage (UHC) achievement was abstracted from the WHO Global Monitoring Report on UHC [17].

### Analytical methods

#### Classifying sources of medical advice

The base is children who were sick with ARI/diarrhoea/fever and sought medical advice outside home. Sources of medical advice are typically categorised in the DHS into broad groups such as ‘public health sector’, ‘private health sector’, ‘other sources’. Each of these broad groupings have several categories depending on type of facility or provider. The public sector sources are typically categorised as ‘government health post’, ‘government health centre, ‘public mobile clinic’, ‘public fieldworker’, ‘public hospital’ or ‘other public sector facility’. Community health worker visits are included. We grouped all these public sources except for ‘public hospital’ into ‘public PCF’. We grouped as ‘private’ sources of care from any private health sector facility, NGO sector facility, or any other non-public source. Thus, every patient visit was assigned to either ‘public PCF’ or ‘public hospital’ or ‘private’ provider.

#### Determinants of PCF use

The Anderson’s behavioural model of health services use identifies contextual and individual predisposing, enabling, and environment factors that facilitate or impede health service use [18]. We incorporate such factors in a generalised linear regression model with logit link function:


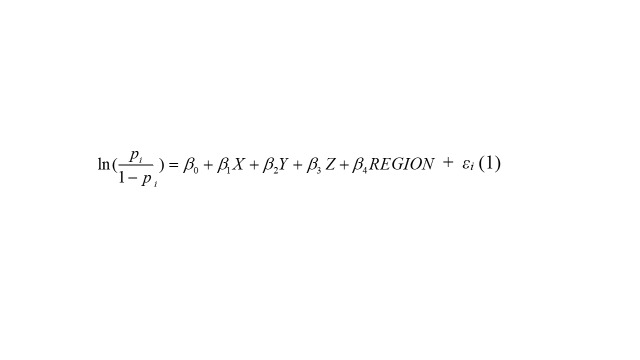
 +  *ε_i_*(1)

where *p* is the proportion of visits to PCFs by ill children with ARI seeking treatment in country i, *X* is a vector of predisposing variables (percentage of population over 65 years, percentage of urban population, primary education complete rate) and their coefficients, *Y* is a vector of enabling variables (per capita GDP, government share in total health expenditure, the density of doctors, and nurses & midwives per 1000 population) and their coefficients, *Z* is the environmental variable (infant mortality rate), REGION indicates region the country is located, and *ε_i_* is the error term.

#### PCF use, universal coverage, and financial protection

We examine the association between high PCF use and achievement on the UHC service coverage index, and catastrophic health expenditures. The UHC service coverage index combines country performance on fourteen indicators drawn from four key areas related to health service coverage: reproductive, maternal, newborn, and child health (RMNCH), infectious diseases, noncommunicable diseases, and service access and capacity [19]. Catastrophic health expenditure is the proportion of a country’s population that has out-of-pocket expenditures in excess of 10% of their income or consumption expenditure (budget). Because the DHS surveys are from different years, we matched the year of survey with the nearest years UHC service coverage index value. However, the latest available catastrophic health expenditure values were used. Countries were classified as ‘low public PCF use’, ‘moderate public PCF use’, or ‘high public PCF use’ if less than 33%, 33–66%, and more than 66% of the ARI treatment visits were to a public PCF, respectively.

The association between public PCF use, UHC coverage and catastrophic health expenditures was examined graphically and using regression analysis. In the regression analysis, for the dependent variable of UHC coverage index we fitted multiple linear regressions, and for the dependent variable of proportion of individuals experiencing catastrophic health expenditures, we fitted generalised linear regression model with logit link function, adjusted for relevant factors - population above 65 years, urban population share, per capita GDP, share of government expenditure in total health spending, doctor and nurse density (Appendix 2 in the **Online Supplementary Document**).

All analyses were conducted using Stata, 17.0 (StataCorp College Station, TX, USA). No ethical review was necessary because publicly available secondary data are used in the analysis. The paper adheres to the STROBE guidelines for reporting observational studies.

## RESULTS

The final analysis included 45 countries, of which, 13 countries were low-income, 26 were lower middle-income, and six were upper middle-income countries. Geographically, two countries were in the Caribbean, one in the middle East, three in central Asia, six in east-Asia, six in South Asia, and the remaining 28 countries in sub-Saharan Africa.

### Use of public PCFs

**Figure 1:**
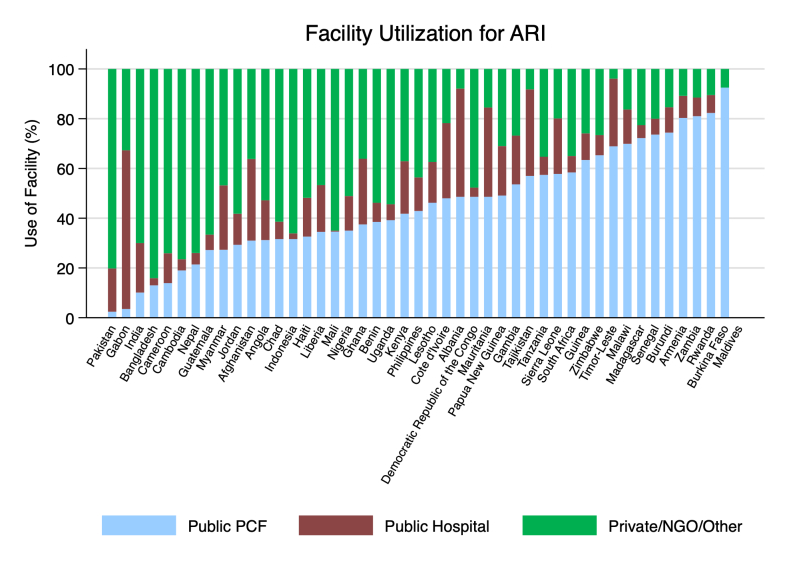
Source of medical advice sought by children under-five years of age for acute respiratory infection (ARI). Source: Authors calculations based on Demographic and Health Surveys (**Online Supplementary Document**).

In most countries (~ 60%) public PCFs received less than half the children seeking outpatient medical advice for ARI, diarrhoea, or fever (**Figure 1**; Appendix 3 in the **Online Supplementary Document**). Use of public PCFs for medical advice varied across countries – in Zambia and Rwanda nearly all ill children who sought medical advice outside their home visited a public PCF, while in South Asia less than 10% of such children visited a public PCF. The median share of medical advice visits for ARI treatment was 43% at public PCFs, compared to 13% at public hospitals and 36% at private providers. Similar distributions are seen for diarrhoea and fever.

Across countries, as the share of medical advice visits to public PCFs declines, the share of visits to private providers increases (**Figure 1**; Appendix 3 in the **Online Supplementary Document**). Notably, public hospitals do not compensate for declines in the share of public PCF visits, though there are exceptions (*e.g.* Tajikistan, Mauritania, Cote d’Ivoire). In general, countries that have high (50% or more medical advice visits) share of visits to public PCFs, have low shares of visits to private providers. The private sector in LMICs typically includes a range of providers – qualified for-profit and not-for profit, and informal providers. Notably, in South Asia, where the share of visits to public PCFs is remarkably low (**Figure 1**), the private sector dominates primary care provision – for example, in Pakistan and Bangladesh around 80%, and in India and Nepal around 70%, of children seeking medical advice for ARI visited a private provider.

### Public PCF use and income

**Figure 2:**
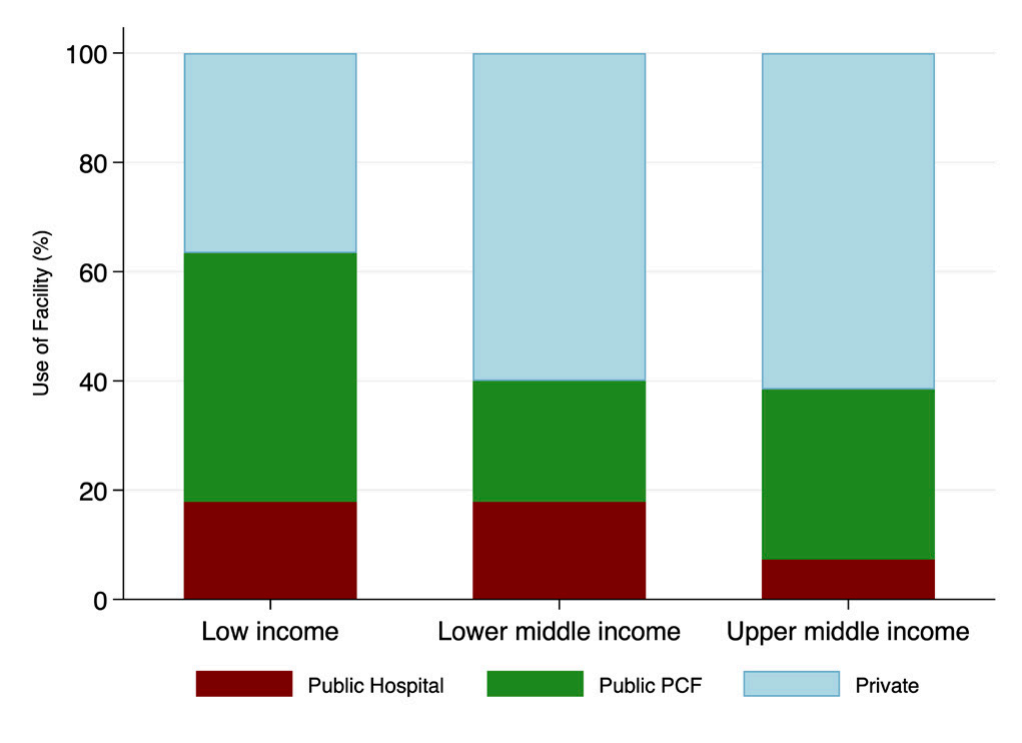
Income group and source of medical advice for children under-five years of age with acute respiratory infection (ARI). Source: Authors calculations based on Demographic and Health Surveys (**Online Supplementary Document**).

**Figure 3:**
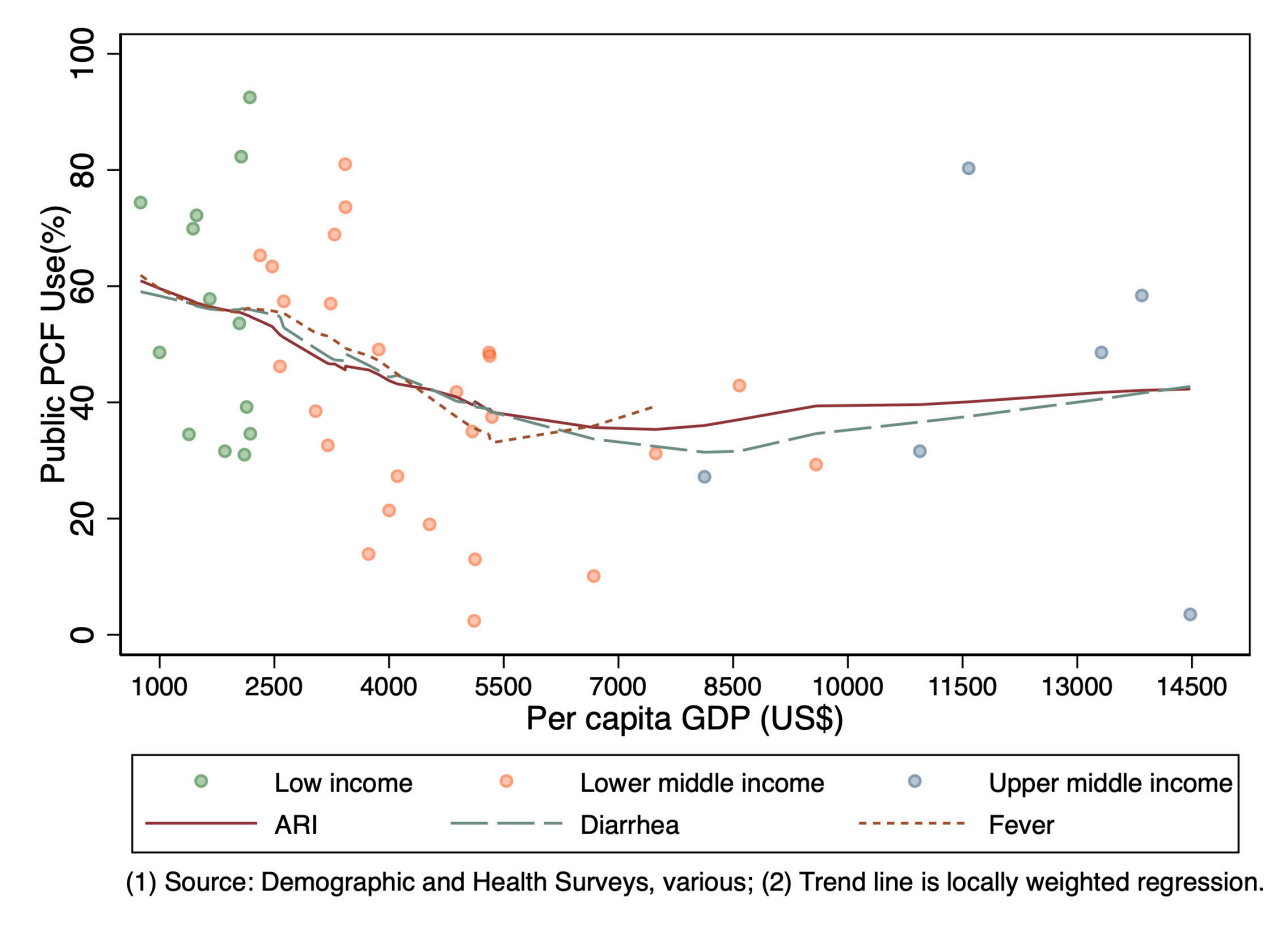
Medical advice sought at public PCFs and per capita GDP [14].

Transitioning from low to middle-income status, a ‘service use transition’ is observed (**Figure 2**, **Figure 3**). This service use transition is characterised by two related trends – decline in the share of patients seeking medical advice at public providers overall and at public PCFs, and a proportionate increase in the share of patients seeking medical advice at private providers. In low-income countries, the largest share of visits was to public PCFs, which declined by around 20 percentage points in lower middle-income countries as private providers increased their share by a similar amount. In upper middle-income countries, the private provider share marginally increases, and within the public sector share, the relative share of public PCFs increased, while that of public hospitals decreased. We note, however, that the upper middle-income country estimates are based on a small sample of six upper middle-income countries.

The ‘service use transition’ is marked by a high share of public PCF in low-income countries, which declines in middle-income countries (**Figure 3**). There is substantial variation in public PCF use along the trend line, especially at lower levels of national income (**Figure 3**). For example, Uganda and Rwanda both have a similar per capita GDP of 2000 USD; however, in Rwanda 82% of ARI visits were treated at public PCFs compared to 40% in Uganda, where the majority of medical advice visits were to private providers (**Figure 1**). This suggests that attributes of national health systems, like the quality and coverage of public PCF services, and private provider presence, are important drivers of variation in public PCF use between countries.

Regression of the proportion of children ill with ARI seeking medical advice at a public PCF on predisposing, enabling, and need factors associated with the Andreson model (Appendix 4 in the **Online Supplementary Document**) indicates that per capita GDP had a significant negative association with public PHF use. A 1% increase in per capita GDP results in a 24-percentage point reduction in the share of children seeking medical advice for ARI at public PCFs (similar elasticities are seen for diarrhoea and fever in children), adjusting for other factors.

### UHC achievement and financial protection

Countries in low, medium and high public PCF use categories are seen across the UHC index range, suggesting that variation in the share of public PCF use is not associated with UHC achievement (Appendix 4, Figure 5 in the **Online Supplementary Document**). On the other hand, expectedly, high public PCF use countries tend to cluster at the lower end of the catastrophic health expenditure axis, indicating that high share of public PCF use is associated with greater financial protection. Regression analysis confirms that variation in public PCF use for children with ARI is not significantly associated with the UHC index, adjusted for other factors (population age above 65%, percentage of urban population, primary school completion, per capita GDP, percentage share of government expenditure in total health spending, doctor and nurse density, and infant mortality) (**Table 1**; Appendix 4 in the **Online Supplementary Document**). However, public PCF use among children with ARI was positively and significantly associated with catastrophic health expenditure (dependent variable), adjusted for other factors (population age above 65%, percentage of urban population, primary school completion, per capita GDP, percentage share of government expenditure in total health spending, UHC index) (**Table 1**; Appendix 4 in the **Online Supplementary Document**). Marginal effects based on the regressions for catastrophic health expenditure indicate that a 10% increase in the use of public PCF is associated with a 1 percentage point reduction in catastrophic health expenditure (**Table 1**).

**Table 1:** Marginal effects of public primary care facility (PCF) use on Universal Health Coverage (UHC) index achievement and catastrophic health expenditure*

Outcome	UHC Index (0–100)	Catastrophic health expenditure (0–1)	Outcome
Marginal effect of public PCF use (95% CI)	0.031 (−0.075, 0.137)	−0.001 (−0.002, −0.0002)	Marginal effect of public PCF use (95% CI)
Observations (countries)	44	44	Observations (countries)

*Marginal effects shown indicates that a percentage point increase in public PCF use is associated with a 3 unit change in the UHC index and a 0.1 percentage point decline in catastrophic health expenditure, controlling for other factors (Appendix 2 in the **Online Supplementary Document**).

## DISCUSSION

This study has three principal findings. First, there appears to be considerable underused capacity of public PCFs in LMICs. In the majority of countries in our sample, public PCFs received less than half the patients seeking medical advice for conditions treatable at the primary care level. Second, economic development in countries is associated with a ‘health service transition’ characterised by two related trends – decline in the share of patients seeking medical advice at public providers overall and at public PCFs, and a proportionate increase in the share of patients seeking medical advice at private providers. Third, high UHC achievement is not associated with public PCF use. However, public PCF use is positively associated with greater financial protection. In combination, these findings have important implications for primary health care policy in low and middle-income countries, particularly those that are in developing economically.

### Service use transition

The ‘service use transition’ marks a reduction in public PCF use. Public PCF use is highest among low-income countries, and declines in middle income countries. A 1% increase in per capita GDP results in a 24-percentage point reduction in the share of public PCFs. Seen another way, if a low-income country with a per capita GDP of 751 USD (*i.e.* average of countries in this group) achieved a per capita GDP of 2544 USD (the average of lower middle-income countries), a 26 percentage point decline in the share of public PCF use is expected. These elasticities indicate that demand for services as public PCFs declines with increased income. Contextual changes associated with economic development, such as higher share of urban populations, reduced public PCF use. However, higher physician density was associated with higher public PCF use.

Primary health care delivery models in LMICs were designed for contexts of rurality, poor connectivity, and lack of access to qualified health care providers (*i.e.* the imperative to bring health services to communities). As countries develop economically these factors change – urbanisation spreads, better and more extensive road networks are built connecting rural communities to towns and cities, and mobility is easier due to improved transportation systems as well as acquisition of personal vehicles. These factors motivate patients to access health care markets and providers, often in the private sector or at higher level facilities, that are farther away than their local public PCF. Further, with growing personal incomes, patients have stronger preferences for better quality services, which often translates to increased demand for private care or services at higher level facilities. That economic development affects patterns of service use has been noted by others. For example, Grépin (2016) finds that the transition from low-income to middle-income status is accompanied by increased private sector use, which then declines in upper-middle income countries presumably because of expansion of social safety nets and government investment in universal health coverage programmes.

### Public PCF use and UHC achievement

The service use transition results in falling share of public PCF use as countries develop economically. A key question is if this change in public PCF use impacts coverage of primary care services and financial protection, key elements of UHC. Our findings indicate that variation in public PCF use is not associated with UHC achievement (as measured by WHO’s UHC index), though it is significantly associated with financial hardship in the population. High levels of UHC achievement are attainable even with low public PCF use because a similar range of services are offered by private providers. As such, private providers substitute for services in public PCFs. However, the widespread use of public PCFs is associated with higher levels of financial protection because public PCFs offer free or highly subsidised primary care services, while health care obtained from private providers is typically paid out-of-pocket by patients in LMICs. Moreover, the largest share of out-of-pocket payments for health care is attributable to primary care services (including medicines) [20]. In countries where public PCF use is low and there is no effective national mechanism for financial protection, expectedly, there will be high levels of financial hardship in the population. For low-income countries that are growing economically and middle-income countries, the fall in public PCF share and increase in private provider share in outpatient visits that characterises the service use transition, makes it imperative to implement programmes for financial protection to protect households from financial hardship.

The agency that public PCFs, and by extension governments, have in influencing health diminishes when communities do not engage with public PCFs for their health needs. When substantial numbers of local patients stay away from public PCFs, the latter have limited information or influence on the health of local communities. In LMICs experiencing the service use transition, a key policy challenge is how to improve their relevance to communities. Clearly, for countries in this situation, there is an urgent need to re-think models of primary care service delivery. One strategy commonly pursued by governments is to expand and strengthen the network of public PCFs. This is unlikely to be successful; studies show that in health markets with large private sector presence, patient often bypass nearby public PCFs even when the public PCFs are located close to their homes, and patients tend to use public PCFs for low severity illnesses [7]. Further, increasing structural or clinical quality of care has only modest effects on increasing public PCF use [9]. Strategies such as empanelment of catchment populations, regular home visits by community health worker can enable public PCFs to engage better with their communities and become more relevant to the health of the population they serve. Another strategy pursued in some countries is to use public funds to strategically purchase health services from private providers [21]. Such strategic purchasing provides financial protection to patients [22–24]. However, it remains to be seen if the transactional nature of purchasing services can enthuse private providers to comprehensively and continuously engage with the health of their communities.

### Limitations

The set of low income, and middle-income countries selected is not a representative sample of these categories. As such, the findings may lack external validity. Second, the observed variation of medical advice sought at public PCFs, public hospitals, and private providers in countries could simply be a function of availability of facilities; this is unlikely to be case everywhere since a sizeable literature has highlighted the issue of bypassing public PCFs. However, we were unable to do any analysis of bypassing in the present study. Further, the DHS surveys record where a sick child sought care but do not identify if it was first contact or usual source of care. Third, because we use 2019 per capita GDP estimates for countries surveyed from 2020–2022, *i.e.* the covid years, there is a temporal mismatch which could bias the associations we report. Fourth, in examining the association between the UHC index and public PCF use, we matched the nearest year of the UHC index and DHS survey year when the same year was not available, this mismatch can bias inferences of the association between UHC index and public PCF use. There are other notable limitations – the DHS surveys, like any standard household survey, record household member self-reports on illness and treatment, which can be potentially biased; the classification of ‘public’ and ‘private’ sources of treatment may not be consistent across countries which can make cross-country comparisons difficult; our analysis of public PCF use is based on care-seeking patterns for illness in children, as such, the patterns reported here may not hold for adults or those with chronic illnesses, which limits generalisability of our conclusions.

Public PCFs can play a key role in influencing the health of communities. Yet, declines in public PCF use as low-income countries get richer or the low use of public PCFs, draws attention to the need to rethink primary health care service delivery models.

## CONCLUSIONS

The changes in care-seeking patterns and use of public PCFs brought about by economic development makes it critical to re-think primary health care service delivery models in transition countries. Future research would do well to focus on the types of interventions or models that can make public PCFs more relevant to community health needs and improve government agency in the health of communities.

## Additional material


Online Supplementary Document
