# Supplementary material for: Use of public primary care facilities, economic development, and the health service transition
Source: J Glob Health. 2026 Apr 10;16:04142. doi: 10.7189/jogh.16.04142 (PMC13067298; doi:10.7189/jogh.16.04142)
Supplement: Online Supplementary Document [file jogh-16-04142-s001.pdf]

Supplement to: Rao KD, Schmidt A, Zhao Y. Use of public primary care facilities, economic development, and the health service transition. J Glob Health. 2026;16:04142.

Appendix 1 Selection of countries with Demographic and Health Surveys

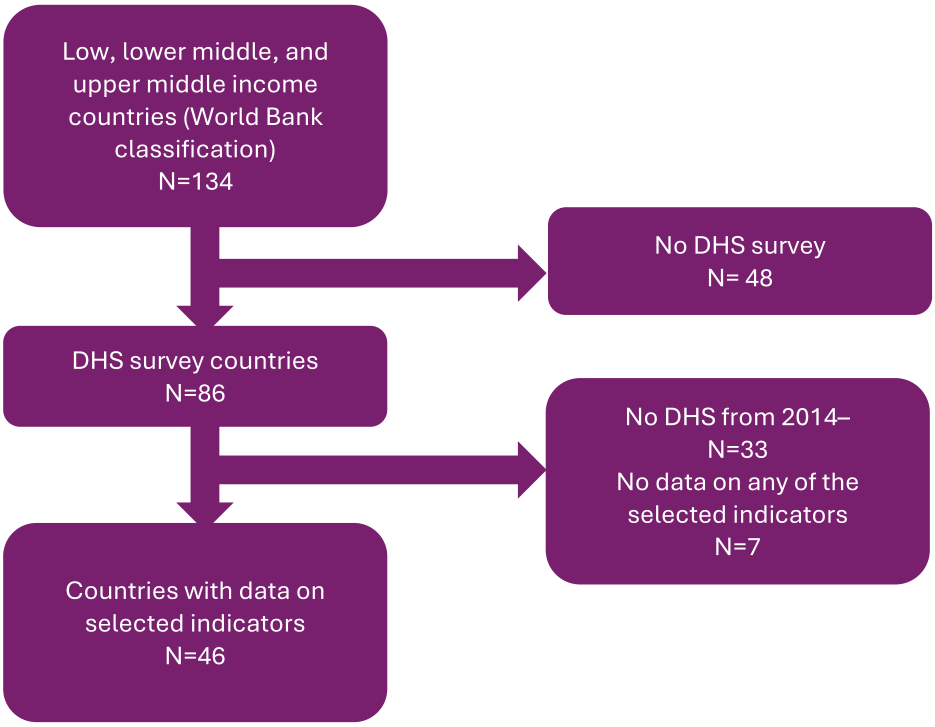

Figure 1: Country inclusion criteria

Country	Region	WB Income	DHS Year
**Afghanistan**	South Asia	Low income	2015
**Albania**	Europe & Central Asia	Upper middle income	2017-18
**Angola**	Sub-Saharan Africa	Lower middle income	2015-16
**Armenia**	Europe & Central Asia	Upper middle income	2015-16
**Bangladesh**	South Asia	Lower middle income	2017-18
**Benin**	Sub-Saharan Africa	Lower middle income	2017-18
**Burkina Faso**	Sub-Saharan Africa	Low income	2021
**Burundi**	Sub-Saharan Africa	Low income	2016-17

**Cambodia**	East Asia & Pacific	Lower middle income	2021-22
**Cameroon**	Sub-Saharan Africa	Lower middle income	2018
**Chad**	Sub-Saharan Africa	Low income	2014-15
**Cote d'Ivoire**	Sub-Saharan Africa	Lower middle income	2021
**Democratic Republic of the Congo**	Sub-Saharan Africa	Low income	2013-14
**Gabon**	Sub-Saharan Africa	Upper middle income	2019-21
**Gambia**	Sub-Saharan Africa	Low income	2019-20
**Ghana**	Sub-Saharan Africa	Lower middle income	2022
**Guatemala**	Latin America & Caribbean	Upper middle income	2014-15
**Guinea**	Sub-Saharan Africa	Lower middle income	2018
**Haiti**	Latin America & Caribbean	Lower middle income	2016-17
**India**	South Asia	Lower middle income	2019-21
**Indonesia**	East Asia & Pacific	Upper middle income	2017
**Jordan**	Middle East & North Africa	Lower middle income	2017-18
**Kenya**	Sub-Saharan Africa	Lower middle income	2022
**Lesotho**	Sub-Saharan Africa	Lower middle income	2014
**Liberia**	Sub-Saharan Africa	Low income	2019-20
**Madagascar**	Sub-Saharan Africa	Low income	2021
**Malawi**	Sub-Saharan Africa	Low income	2015-16
**Maldives**	South Asia	Upper middle income	2016-17
**Mali**	Sub-Saharan Africa	Low income	2018
**Mauritania**	Sub-Saharan Africa	Lower middle income	2019-21
**Myanmar**	East Asia & Pacific	Lower middle income	2015-16
**Nepal**	South Asia	Lower middle income	2022

**Nigeria**	Sub-Saharan Africa	Lower middle income	2018
**Pakistan**	South Asia	Lower middle income	2017-18
**Papua New Guinea**	East Asia & Pacific	Lower middle income	2016-18
**Philippines**	East Asia & Pacific	Lower middle income	2022
**Rwanda**	Sub-Saharan Africa	Low income	2019-20
**Senegal**	Sub-Saharan Africa	Lower middle income	2019
**Sierra Leone**	Sub-Saharan Africa	Low income	2019
**South Africa**	Sub-Saharan Africa	Upper middle income	2016
**Tajikistan**	Europe & Central Asia	Lower middle income	2017
**Tanzania**	Sub-Saharan Africa	Lower middle income	2022
**Timor-Leste**	East Asia & Pacific	Lower middle income	2016
**Uganda**	Sub-Saharan Africa	Low income	2016
**Zambia**	Sub-Saharan Africa	Lower middle income	2018
**Zimbabwe**	Sub-Saharan Africa	Lower middle income	2015

Table 1: Included countries

Appendix 2: Indicator descriptions

Table 2: Indicators included in analysis

Indicator	Description	Data Source
**Income**	According to World Bank income classification;<br>Low income: ≤$1,135 GNI per capita<br>Lower middle income: $1,136-4,465 GNI per capita	World Bank
**Gross Domestic Product (GDP)**	GDP per capita, PPP (constant 2017 international $, matched to DHS survey year)	World Bank
**Government Health Expenditure**	Domestic general government health expenditure (% of GDP, matched to DHS survey year)	World Bank
**Catastrophic Health Spending 10%**	Incidence of catastrophic health spending (%) at 10% of household total consumption or income	WHO Global Monitoring Report on UHC
**Catastrophic Health Spending 25%**	Incidence of catastrophic health spending (%) (at 25% of household total consumption or income)	WHO Global Monitoring Report on UHC
**Out of Pocket Expenditure**	Out-of-pocket expenditure (% of current health expenditure, matched to DHS survey year)	World Bank
**UHC Service Coverage Index**	An index composed of 14 tracer indicators of health service coverage	WHO
**ARI Public Hospital**	Of children with symptoms of ARI for whom advice or treatment was sought, children receiving from a government hospital	StatCompiler
**ARI Public PHC**	Of children with symptoms of ARI for whom advice or treatment was sought, children receiving from government health center, government health post, public mobile clinic, public fieldworker, or other public sector facility	StatCompiler
**ARI Private/Other**	Of children with symptoms of ARI for whom advice or treatment was sought, children receiving from a private medical sector facility, NGO sector facility, or any other source	100 – (ARI Public Hospital + ARI

		Public PHC)
**Diarrhea Public Hospital**	Among children with diarrhea for whom advice or treatment was sought, children receiving from a government hospital	StatCompiler
**Diarrhea Public PHC**	Of children with diarrhea for whom advice or treatment was sought, children receiving from government health center, government health post, public mobile clinic, public fieldworker, or other public sector facility	StatCompiler
**Diarrhea Private/Other**	Of children with symptoms of ARI for whom advice or treatment was sought, children receiving from a private medical sector facility, NGO sector facility, or any other source	100 – (Diarrhea Public Hospital + Diarrhea Public PHC)
**Fever Public Hospital**	Of children with fever for whom advice or treatment was sought, the source was a government hospital	StatCompiler
**Fever Public PHC**	Of children with fever for whom advice or treatment was sought, children receiving from government health center, government health post, public mobile clinic, public fieldworker, or other public sector facility	StatCompiler
**Fever Private/Other**	Of children with fever for whom advice or treatment was sought, children receiving from a private medical sector facility, NGO sector facility, or any other source	100 – (Fever Public Hospital + Fever Public PHC)

Appendix 3: Figures

Figure 1: Source of medical advice sought by children under-five years of age for diarrhea

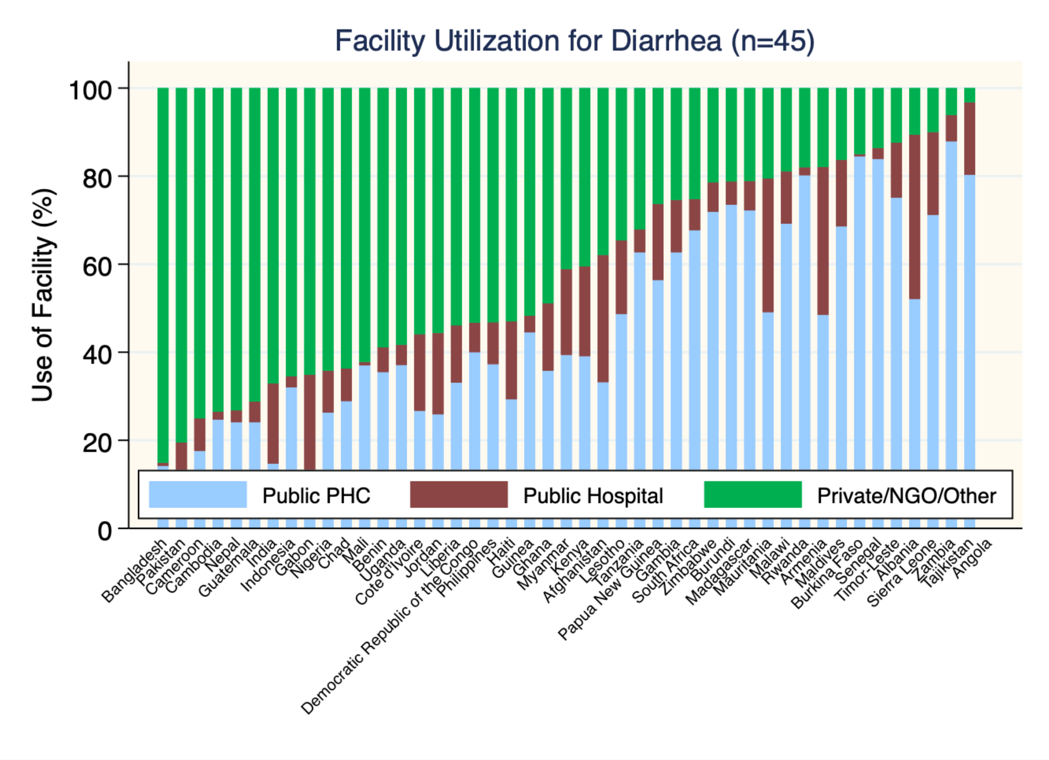

Figure 2: Source of medical advice sought by children under-five years of age for fever

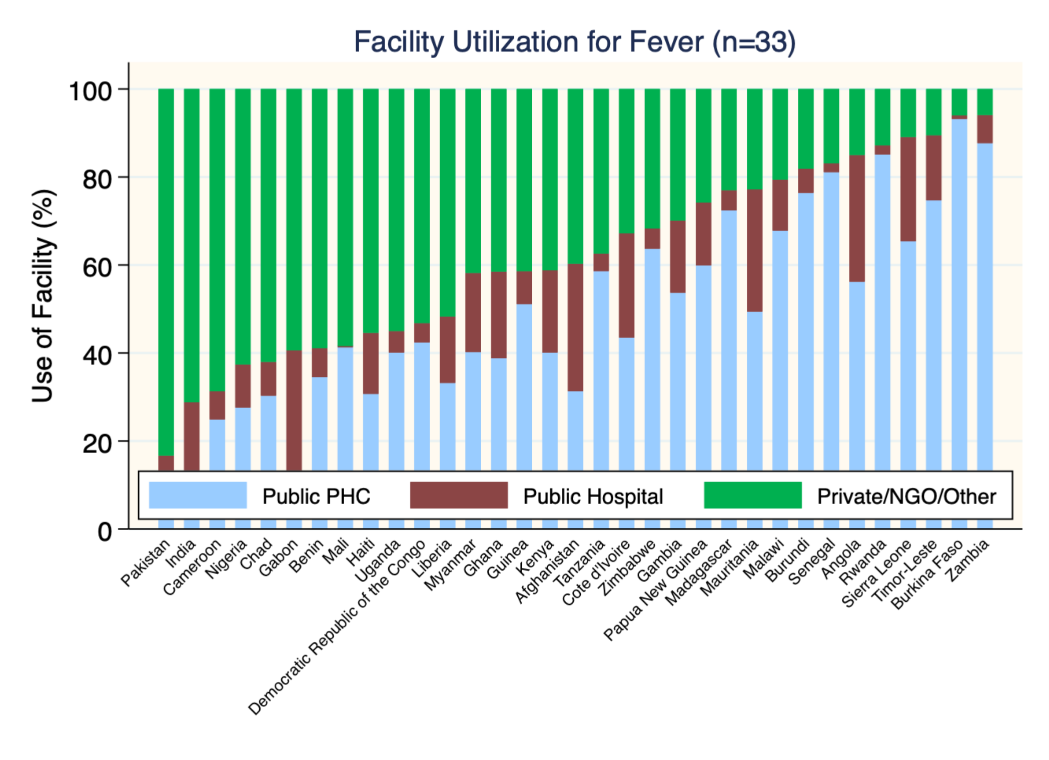

Figure 3: Income group and source of medical advice for children under-five years of age with acute respiratory infection (diarrhea)

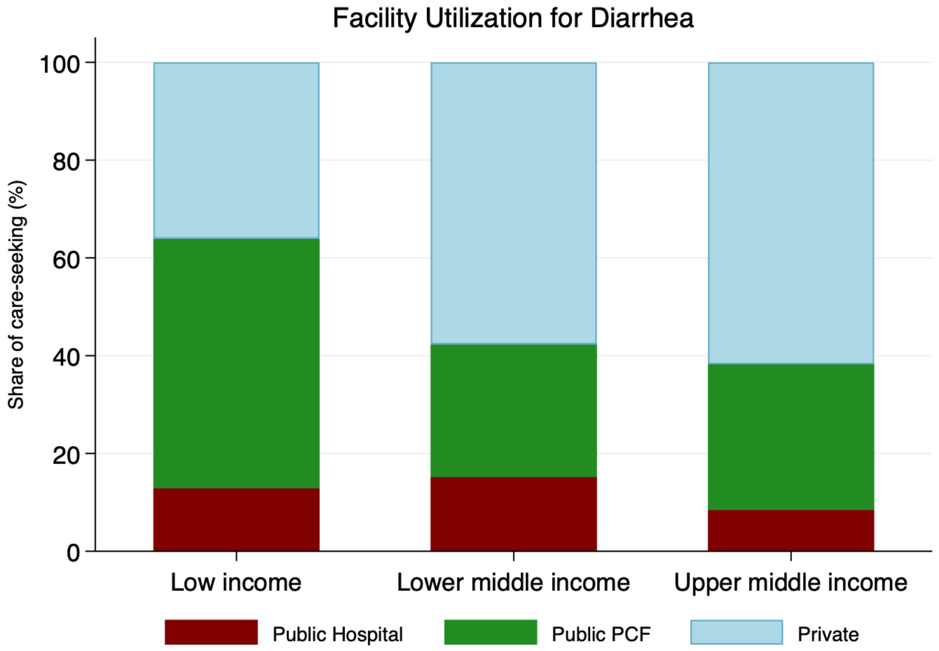

Figure 4: Income group and source of medical advice for children under-five years of age with acute respiratory infection (fever)

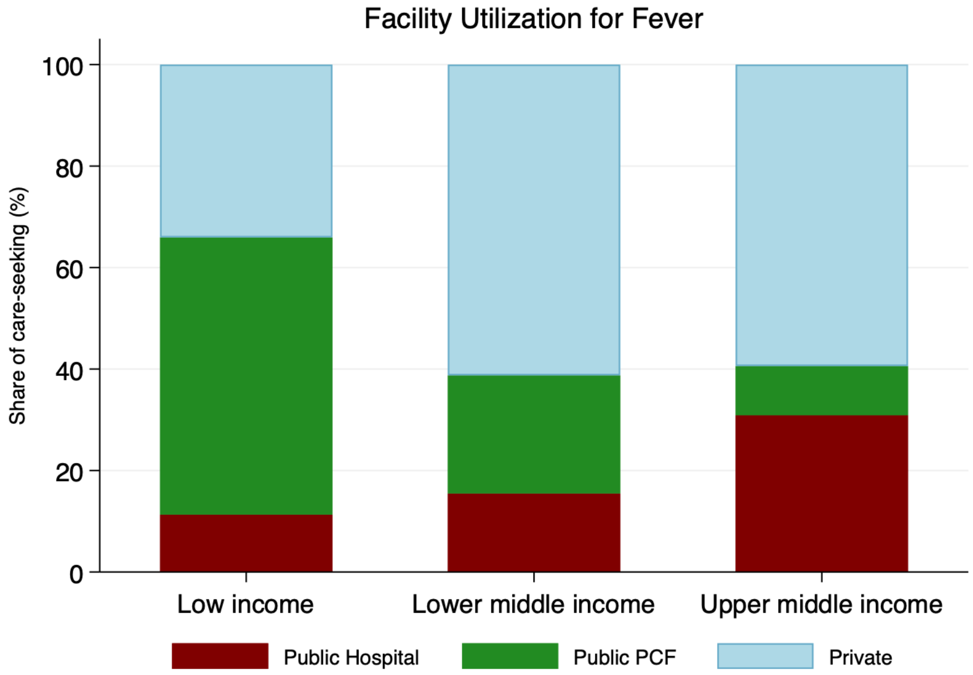

Figure 5 **Figure 3.** UHC achievement, financial protection and public PCF use.

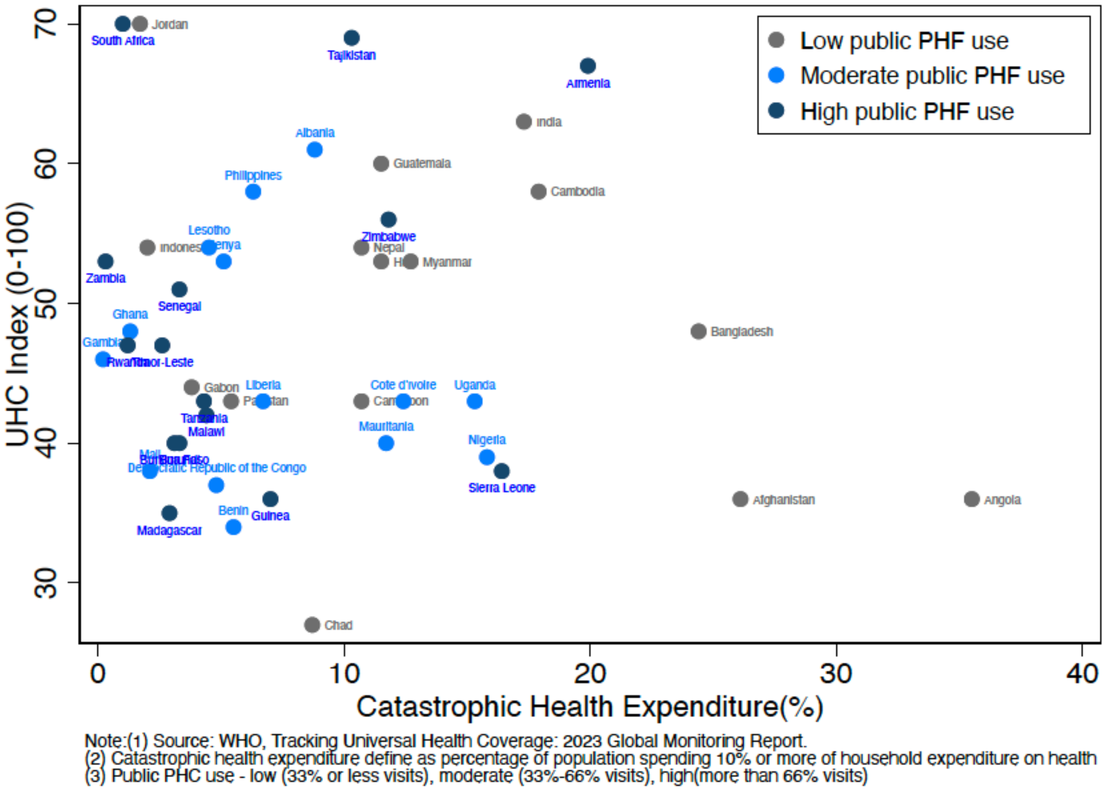

Source: WHO, Tracking Universal Health Coverage: 2023 Global Monitoring Report. Available at: https://www.who.int/publications/i/item/9789240080379.

Appendix 4: Regression Results

Table 1: Descriptive statistics

Table 1: Odds ratio from GLM regression of the proportion children with ARI using PCF on per capita GDP and other variables.

Models for PCF use

	1		2	
lngdp	0.605	**	0.362	***
	[0.4, 0.9]		[0.2, 0.7]	
Population 65years and above (%)			1.052	
			[0.9, 1.2]	
Population urban (%)			1.001	
			[1.0, 1.0]	
Primary school completion rate(%)			1.005	
			[1.0, 1.0]	
Government share in total health expenditure (%)			1.012	
			[1.0, 1.0]	
Physicians per 1000 population			1.142	
			[0.6, 2.1]	
Nurses & midwifes per 1000 population			1.014	
			[0.8, 1.3]	
lnimr			0.728	
			[0.3, 1.6]	
Intercept	50.540	**	3596.133	***
	[2.0,1305.1]		[10.0, 1.3e+06]	
Number of observations	45		45	
Log pseudolikelihood	8.07		11.49	
AIC	-0.27		-0.11	

*** p<.01, ** p<.05, * p<.1

Table 2: Marginal effect of per capita GDP

	dy/dx	Delta-method std. err.	z	P>|z|	[95% conf. interval]	
lngdp	-.2512636	.0796556	-3.15	0.002	-.4073856	-.0951415

Table 3: Linear regression of UHC index on PCF use among children with ARI (ARIPHC)

Table 4: Odds ratio from GLM regression of catastrophic health expenditure on PCF use among children with ARI.
